# Lumbo-Costo-Vertebral Syndrome with Congenital Lumbar Hernia

**Published:** 2014-04-01

**Authors:** Lucky Gupta, Tariq Ahmed Mala, Rahul Gupta, Shahid Amin Malla

**Affiliations:** Department of Surgery,ASCOMS and Hospital Jammu (J and K) India-180017; Department of Surgery,ASCOMS and Hospital Jammu (J and K) India-180017; Department of Paediatric Surgery, SPMCHI, SMS Hospital, Jaipur Rajasthan India; S.S. Medical College Rewa (M.P) India

**Keywords:** Congenital lumbar hernia, Hemivertebra, Absent ribs, Meshplasty

## Abstract

Lumbo-costo-vertebral syndrome (LCVS) is a set of rare abnormalities involving vertebral bodies, ribs, and abdominal wall. We present a case of LCVS in a 2-year-old girl who had a progressive swelling over left lumbar area noted for the last 12 months. Clinical examination revealed a reducible swelling with positive cough impulse. Ultrasonography showed a defect containing bowel loops in the left lumbar region. Chest x-ray showed scoliosis and hemivertebrae with absent lower ribs on left side. Meshplasty was done.

## INTRODUCTION

LCVS consists of abnormal vertebral bodies with abdominal musculature wall defects. Most commonly they appear through superior lumbar triangle of Grynfeltt, and rarely through inferior lumbar triangle of Petit. It may be present at birth or noticed in older age groups [1]. Lower backache is the most common symptom although small hernias may be asymptomatic or present with avisible swelling which becomes more prominent during crying. We herein report a case of LCVS on account of its rarity.

## CASE REPORT

A 2-year-old girl presented with a swelling in left lumbar region noticed by parents one year back. The swelling gradually increased in size. It use to become more prominent on crying. On examination, there was a globular painless swelling (5cm x8cm)which was soft, reducible, and present below the left costal margin lateral to the dorso-lumbar spine (Fig. 1). X-rays of the chest showed D8-L1hemivertebrae, scoliosis with convexity to the right and absent 10th, 11th, and 12th ribs (Fig. 2).Ultrasound abdomen showed a hernial defect in the left lumbar region measuring 4.5cm x 2.9cmwith presence of bowel loops. Operative findings showed hernial sac containing small intestines with absent lower ribs. Hernial sac reduced and mesh was placed in the defect. Postoperative recovery was uneventful. At 1-year follow-up, there was no recurrence.

**Figure:**
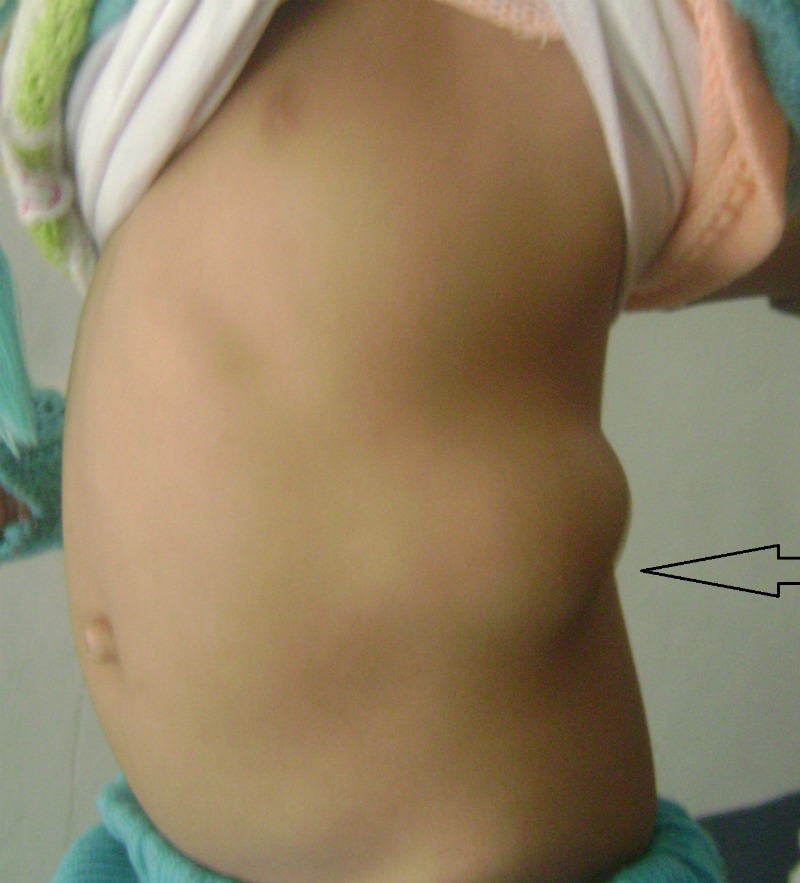
Figure 1:Lumbar hernia

**Figure:**
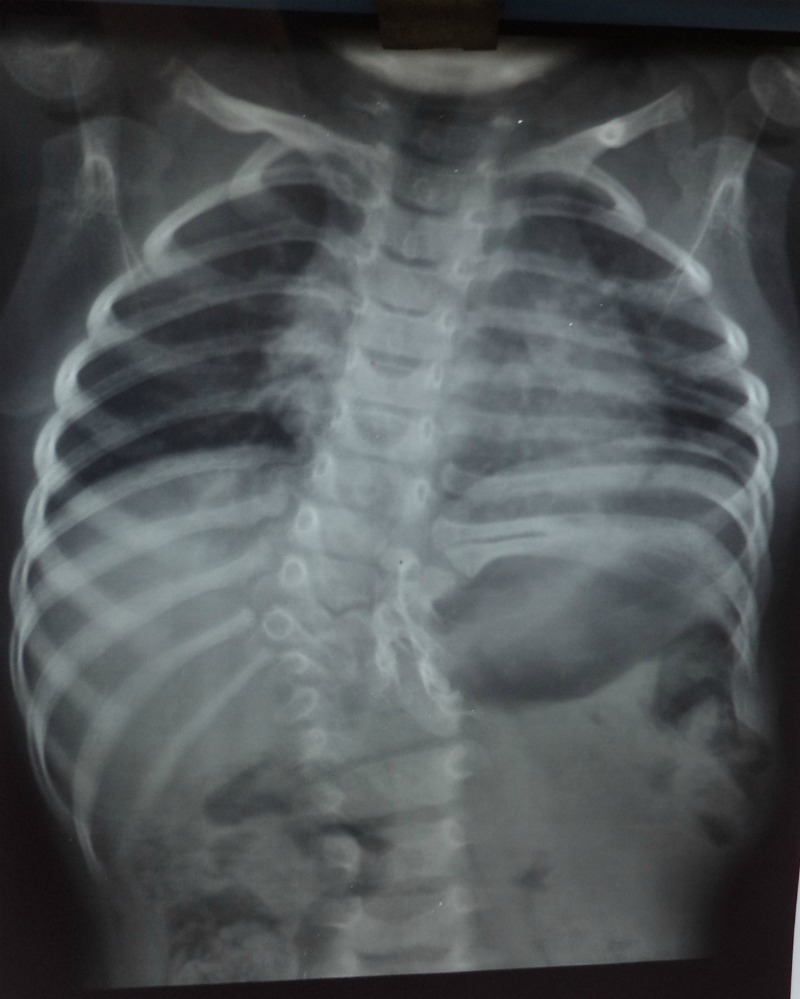
Figure 2:Showing vertebral and rib anomalies.

## DISCUSSION

LCVS was first described by Touloukian in 1972; he believed that these defects are due to a single somatic defect occurring during 3-5 weeks of gestational age, resulting in defective formation of vertebral bodies, ribs, and abdominal wall musculature [2]. Small hernias may remain asymptomatic and rarely present as acute bowel obstruction.[3]Computed tomography (CT) or magnetic resonance imaging (MRI) are investigations of choice to confirm the diagnosis, know hernia contents, and muscular trophies [4]. Contents of hernial sac are usually small or large intestine, mesentery, omentum, appendix, caecum, stomach, ovary, spleen or kidney rarely [1]. In our case, the hernial sac content was small bowel. The goal of repair is to close the defect and built a strong and elastic abdominal wall. Wide variety of materials has been used to close the defect [1]. The simple tissue approximation-closure can be sufficient for small defects while larger defect may need meshplasty as done in the index case. The surgical correction should be done early when the size of the defect is small. Similarly in our patient, the parents reported that the defect had progressively increased in diameter over a year. The defect can be repaired either by open surgery or laparoscopic methods [5].

## Footnotes

**Source of Support:** Nil

**Conflict of Interest:** None declared

